# Effects of Synbiotic Supplementation on Bone Turnover Markers in Kidney Transplant Recipients: A Randomized Controlled Trial

**DOI:** 10.1002/fsn3.72117

**Published:** 2026-07-14

**Authors:** Zeinab Karimi, Hadi Tabibi, Mohsen Nafar, Shiva Samavat, Ahmad Firouzan, Mehdi Hedayati

**Affiliations:** ^1^ Faculty of Nutrition and Food Technology, National Nutrition and Food Technology Research Institute Shahid Beheshti University of Medical Sciences Tehran Iran; ^2^ Department of Clinical Nutrition & Dietetics, Faculty of Nutrition and Food Technology, National Nutrition and Food Technology Research Institute Shahid Beheshti University of Medical Sciences Tehran Iran; ^3^ Chronic Kidney Disease Research Center, Shahid Labbafinejad Medical Center Shahid Beheshti University of Medical Sciences Tehran Iran; ^4^ Department of Nephrology Shahid Beheshti University of Medical Sciences Tehran Iran; ^5^ Cellular and Molecular Endocrine Research Center, Research Institute for Endocrine Sciences Shahid Beheshti University of Medical Sciences Tehran Iran

**Keywords:** bone turnover, C‐telopeptide, kidney transplant recipients, osteocalcin, osteoprotegerin, synbiotics

## Abstract

Bone disorders are common in kidney transplant (KT) recipients. This research explores the impact of synbiotic supplementation on markers related to bone turnover in these patients. In a randomized controlled trial, 46 KT recipients were allocated at random to either a synbiotic group or a placebo group. Those in the synbiotic group received two capsules daily for a duration of 12 weeks, while the placebo group was given an equivalent placebo. The study measured several bone resorption markers, including C‐telopeptide and receptor activator of nuclear factor kappa B ligand (RANKL), as well as serum levels of the bone formation marker osteocalcin and the bone resorption inhibitor osteoprotegerin. The findings revealed that serum levels of C‐telopeptide were significantly lower in the synbiotic group compared to the placebo group (*p* = 0.014). There was a significant increase in osteocalcin levels within the synbiotic group compared to the placebo group (*p* = 0.041). Serum concentrations of RANKL and osteoprotegerin decreased significantly in both the synbiotic and placebo groups compared to baseline. However, the change in serum concentrations of RANKL and osteoprotegerin did not show a significant difference between the two groups. Throughout the study period, no significant changes were observed in serum iPTH, calcium, phosphorus, or 25‐hydroxycholecalciferol levels within each group. In conclusion, synbiotic supplementation decreases serum C‐telopeptide, a marker of bone resorption, and increases serum osteocalcin, a marker of bone formation, in KT recipients.

## Graphical abstract

This study shows that synbiotic supplementation for 12 weeks decreases serum C‐telopeptide, a marker of bone resorption, and increases serum osteocalcin, a marker of bone formation, in KT recipients.

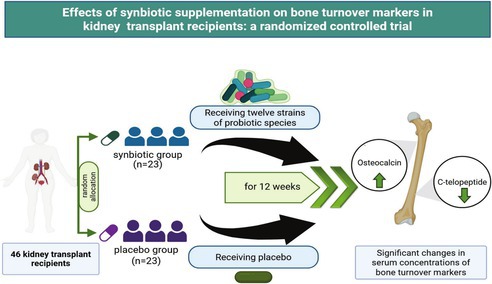

## 1 Introduction

Bone disorders are common in kidney transplant (KT) recipients (Khairallah and Nickolas 2022), leading to bone fractures that occur five times more frequently than in the general population, as well as vascular calcification, cardiovascular diseases, and increased mortality (Khairallah and Nickolas 2022; Molinari et al. 2022). These disorders can stem from several factors, including pre‐existing bone diseases, the use of immunosuppressive glucocorticoid medications such as prednisolone, hyperparathyroidism resulting from poor function of the transplanted kidney, vitamin D deficiency, and severe pre‐transplant hyperparathyroidism (DiCeco et al. 2002; Khairallah and Nickolas 2022; Teh et al. 2024). Additionally, intestinal dysbiosis occurs in these patients (García‐Martínez et al. 2023; Medina and Aykut 2024; Salvadori and Tsalouchos 2021), which may contribute to the development of bone disorders (Xu et al. 2020; Chu et al. 2025). Post‐transplant bone disorders are characterized by increased serum levels of parathyroid hormone (PTH), calcium, and fibroblast growth factor 23 (FGF‐23), as well as decreased levels of phosphorus and vitamin D (Khairallah and Nickolas 2022; Teh et al. 2024). These bone disorders can increase the risk of fractures, vascular calcification, cardiovascular diseases, and increased mortality (Teh et al. 2024; Khairallah and Nickolas 2022; Elder 2023).

Some studies conducted on non‐transplant individuals indicate that probiotics and synbiotics can reduce serum C‐telopeptide concentration, a marker of bone resorption (Jafarnejad et al. 2017; Vanitchanont et al. 2024). According to the available literature, no studies in this field have yet been conducted in KT recipients. Given that the administration of probiotic or synbiotic supplements in transplant recipients has not resulted in any adverse effects and has even been shown to reduce various infections (Zhang et al. 2013), this study aims to investigate the effects of synbiotic supplementation on bone turnover markers in KT recipients.

## 2 Materials and Methods

### 2.1 Participants

This study was a randomized, double‐blind, placebo‐controlled trial, taking place from December 2023 to May 2024. To achieve a statistical power of 80% (1 − *β*) with an *α* level of 0.05, we calculated a minimum required sample size of 19 participants per group to identify a difference of 0.8 ng/mL in serum osteocalcin levels through two‐tailed testing. The pooled standard deviation utilized for this estimation was 0.88 ng/mL, obtained from a previous study (Jafarnejad et al. 2017). A total of 48 KT recipients, aged between 22 and 73 years, were recruited from the Labbafi Nejad Hospital Clinic in Tehran, Iran. The inclusion criteria specified that participants must have undergone kidney transplantation for over 1 year with stable graft function (serum creatinine levels maintained below 2.5 mg/dL for the past 3 months), be at least 18 years old, and possess a body mass index (BMI) of 25 kg/m^2^ or greater.

In our study, participants with a BMI ≥ 25 kg/m^2^ were selected because overweight and obesity are prevalent in KT recipients, and they are more likely to show changes in bone markers as a result of alterations in inflammatory markers.

Individuals with inflammatory diseases, infectious diseases, and diarrhea within the last 3 months, as well as those who had been using probiotic or synbiotic supplements or consuming probiotic‐containing foods were excluded from participation in the study.

The study was conducted in accordance with the guidelines established in the Declaration of Helsinki. Prior to participation, all subjects provided their written informed consent. The protocol for the study received approval from the Ethics Committee at the National Nutrition and Food Technology Research Institute in Iran (Ethical Code: IR.SBMU.nnftri.Rec.1402.051). Additionally, this clinical trial has been registered on the ClinicalTrials.gov website under the identifier NCT06160830.

### 2.2 Study Design

Participants were categorized according to their diabetes status and serum creatinine levels before being randomly allocated to either a synbiotic group or a placebo group. Block randomization, with a size of four and a 1:1 allocation ratio, was employed for this process. The assignment of participants to groups was determined through simple random sampling of the blocks. A qualified dietitian performed the block randomization procedure. The synbiotic group received two capsules of LactoCare containing 1 × 10^9^ CFU each, administered at breakfast and dinner over a period of 12 weeks, while the placebo group received two identical placebo capsules. Each capsule comprised 12 different strains of probiotic species, including *Lactobacillus rhamnosus* , *Lactobacillus casei* , *Lactobacillus acidophilus* , *Lactobacillus bulgaricus* , *Lactobacillus plantarum* , *Lactobacillus gasseri* , *Lactobacillus helveticus* , *Bifidobacterium lactis* , *Bifidobacterium breve, Bifidobacterium longum, Bifidobacterium bifidum*, and *Streptococcus thermophilus* . Additionally, each capsule contains 21 mg of fructo‐oligosaccharides as a prebiotic, along with cellulose, lactose, maltodextrin, sodium starch, magnesium stearate, talc, and aerosol. The placebo capsules contained all ingredients found in the synbiotic capsules, except for the probiotics and fructo‐oligosaccharides. The synbiotic and placebo capsules were both produced by Zist Takhmir Pharmaceutical Company located in Tehran, Iran. In this study, a qualified dietitian managed the blinding process to ensure that neither the participants nor the researchers were aware of the group assignments.

All participants in both groups were either overweight or obese and were in the chronic post‐transplant phase. They were placed on a calorie‐restricted diet specifically designed for this phase, in conjunction with either a synbiotic supplement or a placebo at the commencement of the study. To determine the total energy requirements for these patients, we calculated each individual's basal energy expenditure using the Mifflin‐St Jeor equation (Ireton‐Jones 2021). This value was subsequently multiplied by a physical activity factor of 1.3 and a thermic effect of food factor of 1.1 (Laquatra 1996). A deduction of 20% was applied to the calculated total energy requirement to account for their overweight or obesity status. To establish the protein requirements for overweight or obese KT recipients, we first calculated their adjusted ideal body weight (AIBW) (Laquatra 1996). The protein requirement was set at 1 g of protein per kg of AIBW for individuals with normal serum creatinine levels (Wilkens et al. 2021), and at 0.75–0.8 g of protein per kg of AIBW for those with elevated serum creatinine levels (Wilkens et al. 2021). Participants were instructed to strictly adhere to the provided diet and to refrain from making any changes to their medications or physical activity during the study period.

### 2.3 Assessments

Following the commencement of the study and the execution of the designated dietary interventions, participants' food consumption was recorded using a 3‐day dietary recall method, which included 2 weekdays and 1 weekend day, at the end of Weeks 1, 6, and 12. The collected data were processed with Nutritionist IV software (N Squared Computing, San Bruno, CA). Additionally, participants' weights were measured both at baseline and again at the conclusion of Week 12.

Blood samples of 7 mL were drawn from each participant after a fasting period of 12–14 h in the morning at baseline and Week 12. The samples were allowed to reach room temperature (20°C–25°C) for 15 min before being centrifuged at 2000 rpm for 10 min. The serum obtained was then divided into smaller portions and stored at −80°C until further analysis. Serum concentrations of C‐telopeptide, a bone resorption marker (Nakashima et al. 2005; Kajarabille et al. 2013), were quantified using enzyme‐linked immunosorbent assay (ELISA) kits from Immunodiagnostic Systems (Boldon, UK). Receptor activator of nuclear factor kappa B ligand (RANKL), another bone resorption marker (Kajarabille et al. 2013; Nakashima et al. 2005), osteocalcin, a bone formation marker (Bandeira et al. 2014), and osteoprotegerin, a bone resorption inhibitor (Kajarabille et al. 2013) were determined using an ELISA kits from ZellBio GmbH (Ulm, Germany). Intact parathyroid hormone (iPTH) concentration was determined by an ELISA kit from Beta Biomed (Rowlett, USA), and 25‐hydroxycholecalciferol was assessed using an ELISA kit from Namira Gene (Karaj, Iran). The intra‐assay coefficients of variation (CVs) for serum C‐telopeptide, RANKL, osteocalcin, osteoprotegerin, iPTH, and 25‐hydroxycholecalciferol were 2%, 6.6%, 7.3%, 5.6%, 6.1%, and 8%, respectively. For measurements of serum phosphorus and calcium, commercial kits from Delta Darman Part (Tehran, Iran) and a Pictus 700 Autoanalyzer (Diatron, Budapest, Hungary) were used. The intra‐assay CVs for serum calcium and phosphorus were 1.5% and 3%, respectively. The primary outcomes of this study were serum C‐telopeptide, RANKL, osteoprotegerin, osteocalcin, iPTH, calcium, phosphorus, and 25‐hydroxycholecalciferol.

The study evaluated patient compliance by requiring each participant to return any unused capsules after completing their consumption. The compliance rate was over 90%.

### 2.4 Statistical Methods

All statistical analyses were conducted using the Statistical Package for the Social Sciences (SPSS Inc., Chicago, IL) for Windows version 23.0. To compare categorical variables between the two groups, a chi‐squared test was applied. Given that all quantitative parameters demonstrated a normal distribution according to the Kolmogorov–Smirnov test, both independent sample *t*‐tests and paired *t*‐tests were utilized for comparisons between groups and within groups, respectively. Dietary parameters were assessed at three different time points throughout the study; therefore, analysis of variance for repeated measures was employed. Results are presented as mean ± standard error of the mean, with a *p* value of ≤ 0.05 considered indicative of a statistically significant difference.

## 3 Results

**FIGURE 1:**
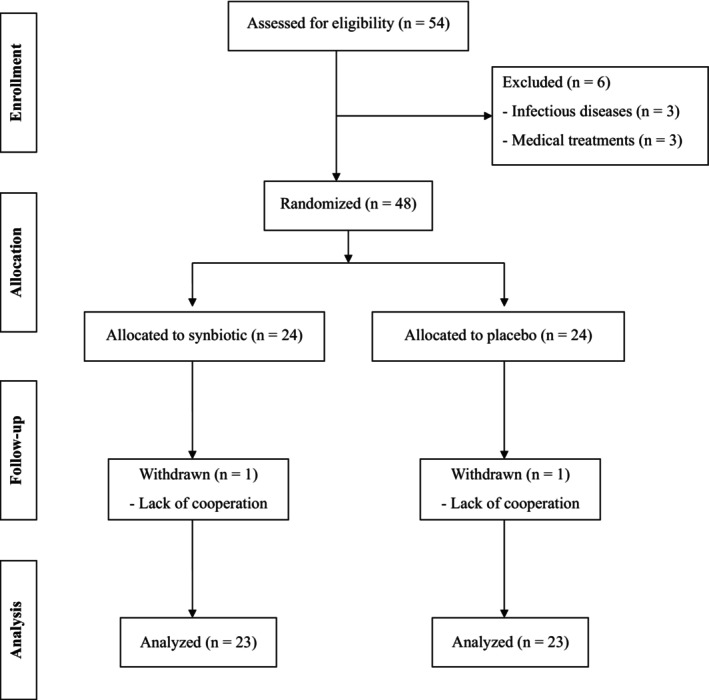
Summary of the patient flow diagram.

Of the 48 KT recipients initially enrolled in the study, one participant from each group was withdrawn due to lack of cooperation (Figure 1). There were no reported adverse events by patients during this investigation.

An analysis of baseline characteristics revealed no statistically significant differences between the two groups (Table 1). Both the synbiotic group (*p* = 0.001) and the placebo group (*p* = 0.02) indicated significant reductions in body weight compared to baseline. In addition, the weight loss in the synbiotic group was significantly higher than that observed in the placebo group (*p* = 0.02; Table 1).

**TABLE 1:** Baseline characteristics of kidney transplant recipients in the synbiotic group and the placebo group.

Characteristics	Synbiotic (*n* = 23)	Placebo (*n* = 23)
Sex		
Men	17 (74%)	14 (61%)
Women	6 (26%)	9 (39%)
Menopausal status		
Premenopausal	4 (67%)	6 (67%)
Postmenopausal	2 (33%)	3 (33%)
Type of immunosuppressive drugs		
Prednisolone + cellcept + tacrolimus	11 (48%)	12 (52%)
Prednisolone + cellcept + cyclosporine	9 (39%)	11 (48%)
Prednisolone + cellcept + rapamycin	1 (4%)	0 (0%)
Prednisolone + tacrolimus + rapamycin	2 (9%)	0 (0%)
Intake of supplements		
Calcium carbonate	12 (52%)	14 (61%)
Vitamin D3	19 (83%)	14 (61%)
ω3 fatty acids	3 (13%)	2 (9%)
Smokers	2 (9%)	1 (4%)
Diabetes	7 (30%)	8 (35%)
Age (year)^a^	52 ± 2	51 ± 2
Kidney transplant vintage (year)^a^	10.5 ± 1.0	11.7 ± 1.5
Serum creatinine (mg/dL)^a^	1.33 ± 0.08	1.50 ± 0.10
Serum urea (mg/dL)^a^	45 ± 5	50 ± 5
Weight (kg)^a^		
Baseline	84.3 ± 2.5	84.0 ± 2.5
Week 12	82.3 ± 2.5^†^	83.2 ± 2.4^‡^
Changes	−2.0 ± 0.4^§^	−0.8 ± 0.2

^a^ Presented as mean ± standard error.

^†^ *p* = 0.001 versus baseline.

^‡^ *p* = 0.02 versus baseline.

^§^ *p* = 0.02 versus placebo.

During Weeks 1, 6, and 12, there were no statistically significant differences observed between the two groups in the mean dietary intake of calcium, phosphorus, magnesium, energy, protein, carbohydrates, and total fat between the two groups. Additionally, these differences were not significant within each group throughout the study duration (Table 2).

**TABLE 2:** Dietary factors of kidney transplant recipients in the synbiotic group and the placebo group.^a^

Factors	Week 1	Week 6	Week 12
Calcium (mg/day)			
Synbiotic	593 ± 54	550 ± 51	534 ± 40
Placebo	573 ± 43	532 ± 39	571 ± 61
Phosphorus (mg/day)			
Synbiotic	790 ± 45	756 ± 59	731 ± 59
Placebo	764 ± 25	789 ± 65	762 ± 51
Magnesium (mg/day)			
Synbiotic	210 ± 13	190 ± 14	182 ± 10
Placebo	209 ± 9	183 ± 18	194 ± 20
Energy (kcal/day)			
Synbiotic	1841 ± 45	1822 ± 124	1811 ± 120
Placebo	1788 ± 56	1775 ± 122	1781 ± 88
Protein (g/day)			
Synbiotic	73 ± 2	65 ± 5	65 ± 5
Placebo	68 ± 2	64 ± 5	66 ± 6
Carbohydrate (g/day)			
Synbiotic	266 ± 8	254 ± 17	259 ± 19
Placebo	258 ± 10	251 ± 18	249 ± 11
Fat (g/day)			
Synbiotic	57 ± 2	63 ± 5	60 ± 5
Placebo	56 ± 2	59 ± 5	61 ± 5

*Note:* All values are presented as mean ± standard error.

^a^ *n* = 23 for all values.

At the end of Week 12, serum concentrations of C‐telopeptide showed a significant decrease in the synbiotic group when compared to baseline levels (*p* = 0.048), whereas no significant changes were noted in the placebo group (Table 3). The reduction in serum C‐telopeptide in the synbiotic group was statistically significant in comparison with the placebo group (*p* = 0.014; Table 3).

**TABLE 3:** Serum concentrations of bone turnover markers, iPTH, calcium, phosphorus, and 25‐hydroxycolecalciferol in the synbiotic group and the placebo group.^a^

Parameters	Baseline	Week 12	Changes^b^	Within‐group *p*
C‐telopeptide (ng/L)				
Synbiotic	467 ± 60	422 ± 51	−45 ± 21	0.048
Placebo	497 ± 74	548 ± 89	51 ± 31	NS
Between‐group *p*	NS	NS	0.014	
RANKL (pg/mL)				
Synbiotic	182 ± 11	162 ± 11	−20 ± 4	0.001
Placebo	232 ± 35	217 ± 36	−15 ± 4	0.001
Between‐group *p*	NS	NS	NS	
Osteocalcin (ng/mL)				
Synbiotic	14 ± 1	15 ± 1	1 ± 0.2	0.001
Placebo	18 ± 2	18 ± 2	0 ± 0.3	NS
Between‐group *p*	NS	NS	0.041	
Osteoprotegerin (ng/mL)				
Synbiotic	1.60 ± 0.09	1.53 ± 0.08	−0.07 ± 0.02	0.009
Placebo	2.08 ± 0.33	1.97 ± 0.33	−0.11 ± 0.04	0.011
Between‐group *p*	NS	NS	NS	
iPTH (pg/mL)				
Synbiotic	61 ± 8	56 ± 8	−5 ± 3	NS
Placebo	80 ± 11	72 ± 10	−8 ± 5	NS
Between‐group *p*	NS	NS	NS	
Calcium (mg/dL)				
Synbiotic	9.2 ± 0.1	9 ± 0.3	−0.2 ± 0.3	NS
Placebo	9.2 ± 0.2	9 ± 0.2	−0.2 ± 0.2	NS
Between‐group *p*	NS	NS	NS	
Phosphorus (mg/dL)				
Synbiotic	4.2 ± 0.1	4.3 ± 0.1	0.1 ± 0.1	NS
Placebo	4.5 ± 0.1	4.6 ± 0.1	0.1 ± 0.1	NS
Between‐group *p*	NS	NS	NS	
25‐hydroxycolecalciferol (ng/mL)				
Synbiotic	41 ± 3	37 ± 3	−4 ± 3	NS
Placebo	45 ± 4	43 ± 5	−2 ± 4	NS
Between‐group *p*	NS	NS	NS	

*Note:* All values are presented as mean ± standard error.

Abbreviations: iPTH, intact parathyroid hormone; NS, nonsignificant; RANKL, receptor activator of nuclear factor kappa B ligand.

^a^ *n* = 23 for all values.

^b^ Changes reflect Week 12 – baseline values.

Serum concentrations of RANKL decreased significantly in both the synbiotic (*p* = 0.001) and placebo (*p* = 0.001) groups at the end of Week 12 compared to baseline values. However, no significant difference was found in RANKL reductions between the two groups (Table 3).

A significant increase in serum osteocalcin was recorded in the synbiotic group at Week 12 compared to baseline (*p* = 0.001), while no significant changes were observed in the placebo group (Table 3). The rise in serum osteocalcin levels in the synbiotic group was statistically significant in comparison with the placebo group (*p* = 0.041; Table 3).

Serum concentrations of osteoprotegerin showed a significant decline in both synbiotic (*p* = 0.009) and placebo (*p* = 0.011) groups at the end of Week 12 in comparison with baseline. However, the reductions were not significantly different between the two groups (Table 3).

Throughout the study, there were no significant changes in serum iPTH, calcium, phosphorus, and 25‐hydroxycholecalciferol within each group (Table 3).

## 4 Discussion

In the present study, serum C‐telopeptide concentration, a marker of bone resorption released from bone due to osteoclast activity (Wheater et al. 2013), significantly decreased in the synbiotic group compared to the placebo group. There were no existing studies on the effects of synbiotic supplementation on serum C‐telopeptide levels in KT recipients for comparison with our results. Consistent with our findings, several studies involving postmenopausal women have shown that probiotic supplementation leads to a reduction in serum C‐telopeptide concentration compared to the placebo group (Jafarnejad et al. 2017; Vanitchanont et al. 2024). Additionally, a meta‐analysis has demonstrated that probiotic supplementation in postmenopausal women is associated with a reduction in serum C‐telopeptide concentration (Wang et al. 2024). However, some research has suggested that probiotic supplementation does not yield a reduction in serum C‐telopeptide among postmenopausal women (Han et al. 2022; Gregori et al. 2024; Harahap et al. 2024). The discrepancies in the effects of probiotics on serum C‐telopeptide concentrations may be attributed to variations in probiotic dosage, the types of strains used, and the initial serum C‐telopeptide concentrations. Research has shown that inflammatory cytokines promote the differentiation and activation of osteoclasts (Alves et al. 2016). Given that probiotics or synbiotics have been shown to reduce inflammation (Ghavami et al. 2021; Bohlouli et al. 2021), the mechanism by which probiotics decrease serum C‐telopeptide concentration may be related to their anti‐inflammatory effects (Wang et al. 2024; Bose and Sharan 2024; Collins et al. 2017). Research indicates that obesity is associated with inflammation (Schleh et al. 2023). Therefore, synbiotics may mitigate inflammation by facilitating weight loss, a result that was also observed in this study.

Short‐chain fatty acids (SCFAs) produced by probiotics in the intestine can inhibit the synthesis of inflammatory cytokines through their inhibitory effects on nuclear factor‐κB and mitogen‐activated protein kinases (Wang et al. 2025). In addition, SCFAs can establish an acidic environment by decreasing the pH level of the intestinal tract, which subsequently enhances calcium absorption (Chen et al. 2022).

Another marker of bone resorption is RANKL, a ligand for receptor activator of nuclear factor kappa‐B (RANK) on osteoclasts, which is synthesized by various cells, including osteoblasts. RANKL promotes the differentiation and activation of osteoclasts (Kajarabille et al. 2013) and inhibits their apoptosis, thereby increasing their lifespan (Yu et al. 2015). In the current study, serum RANKL concentration significantly decreased in both the synbiotic and placebo groups; however, no significant difference was observed in the reduction of serum RANKL concentration between the two groups. Consequently, synbiotic supplementation had no effect on serum RANKL concentration when compared to the placebo. To date, there were no available studies on the effects of synbiotics on serum RANKL concentration in KT recipients, making it impossible to compare their results with those of the present study. Nonetheless, consistent with our findings, some studies have indicated that probiotics have no effect on serum RANKL concentration in postmenopausal women without kidney diseases (Wang et al. 2024; Jafarnejad et al. 2017).

In the present study, serum osteocalcin concentration, a marker of bone formation (Wheater et al. 2013), significantly increased in the synbiotic group compared to the placebo group. To date, no studies have been conducted on the effects of synbiotics or probiotics on serum osteocalcin concentration in KT recipients, which limits our ability to compare our results with those of other research. However, some research in postmenopausal women without kidney diseases has shown that probiotics do not affect serum osteocalcin concentration (Jafarnejad et al. 2017; Han et al. 2022). Additionally, a meta‐analysis indicated that probiotic supplementation has no effect on serum osteocalcin concentration in postmenopausal women (Wang et al. 2024). The discrepancies regarding the effects of probiotics on serum osteocalcin may be due to differences in dosage and strains of probiotics administered, as well as variations in baseline serum osteocalcin concentration. Research has shown that certain inflammatory cytokines inhibit the differentiation and activation of osteoblasts (Alves et al. 2016; Xu et al. 2023). Given that probiotics or synbiotics may reduce inflammation (Bohlouli et al. 2021), the mechanism by which they may increase serum osteocalcin concentration could be attributed to their anti‐inflammatory effects. Studies have shown that obesity is linked to inflammation (Schleh et al. 2023). Consequently, synbiotics may reduce inflammation by promoting weight reduction, a finding also observed in this study.

Osteoprotegerin is produced by various cells, including osteoblasts, and functions as a bone resorption inhibitor. It prevents bone resorption by binding to RANKL and blocking its attachment to RANK. This inhibition of osteoclast activation helps prevent bone loss (Kajarabille et al. 2013). In the current study, serum OPG concentrations significantly decreased in both the synbiotic and placebo groups; however, no significant difference was observed in the reduction of serum OPG concentrations between the two groups. Therefore, synbiotic supplementation did not affect serum OPG concentrations compared to the placebo. To date, no studies have investigated the effect of synbiotics on serum OPG concentrations in KT recipients, making it impossible to compare our results with those of other research. Nevertheless, consistent with our findings, some research has indicated that probiotics have no effect on serum OPG concentrations in postmenopausal women without kidney diseases (Wang et al. 2024; Jafarnejad et al. 2017).

In our study, serum iPTH, calcium, phosphorus, and 25‐hydroxycolecalciferol were confounding variables that did not show statistically significant differences between the two groups. Therefore, changes in bone markers, including serum C‐telopeptide, RANKL, osteocalcin, and OPG cannot be attributed to these confounding variables.

The limitations of our study included the absence of gut microbiota analysis, the lack of serum testosterone measurement, the relatively small sample size, limited power for certain bone markers that did not show statistically significant differences, and the lack of direct assessment of the mechanisms underlying the synbiotic effects, such as measuring blood SCFAs.

## 5 Conclusion

Synbiotic supplementation decreases serum C‐telopeptide, a marker of bone resorption, and increases serum osteocalcin, a marker of bone formation in KT recipients.

## Author Contributions

**Hadi Tabibi:** conceptualization, investigation, methodology, project administration, supervision, writing – original draft, writing – review and editing. **Mohsen Nafar:** conceptualization, investigation, writing – review and editing. **Mehdi Hedayati:** conceptualization, methodology, investigation, writing – review and editing. **Ahmad Firouzan:** conceptualization, investigation, writing – review and editing. **Zeinab Karimi:** conceptualization, investigation, methodology, writing – original draft, writing – review and editing, project administration. **Shiva Samavat:** conceptualization, investigation, writing – review and editing.

## Funding

The authors have nothing to report.

## Conflicts of Interest

The authors declare no conflicts of interest.

## Data Availability

The data that support the findings of this study are available from the corresponding author upon reasonable request.
